# Supplementary material for: Asymmetric cryo-EM reconstruction of phage MS2 reveals genome structure in situ
Source: Nat Commun. 2016 Aug 26;7:12524. doi: 10.1038/ncomms12524 (PMC5007439; doi:10.1038/ncomms12524)
Supplement: Supplementary Information — Supplementary Figures 1-4 [file ncomms12524-s1.pdf]

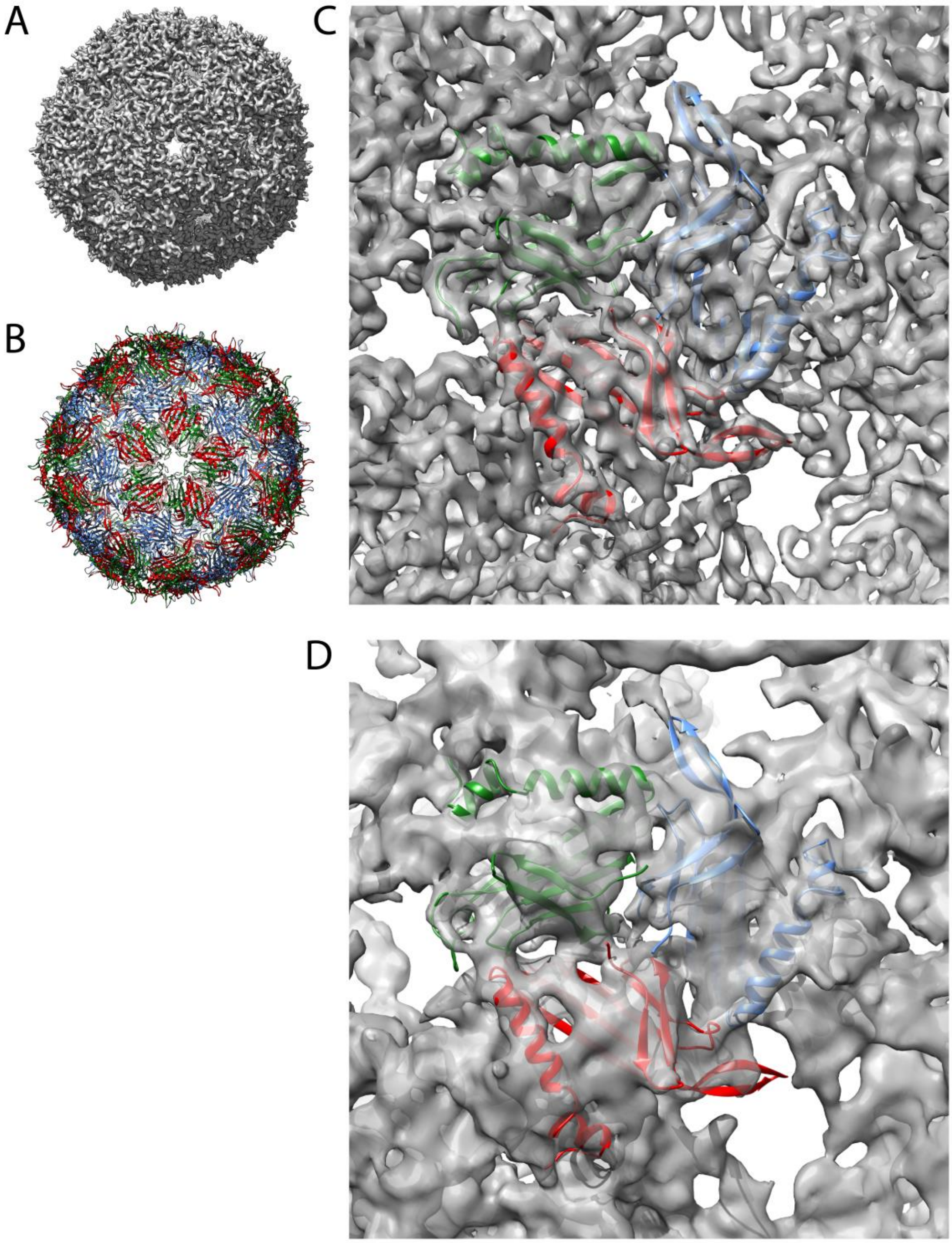

Supplementary Figure 1. Symmetric and asymmetric MS2 density maps.
Icosahedral cryo-EM reconstruction at 4.1 Å of full MS2 virion (a), icosahedral model of X-ray structure of capsid protein (pdb: 2MS2) (b) and fits of X-ray structure (coloured) in EM densities (grey) of the icosahedrally symmetrized map (c) and unsymmetrized map (d).

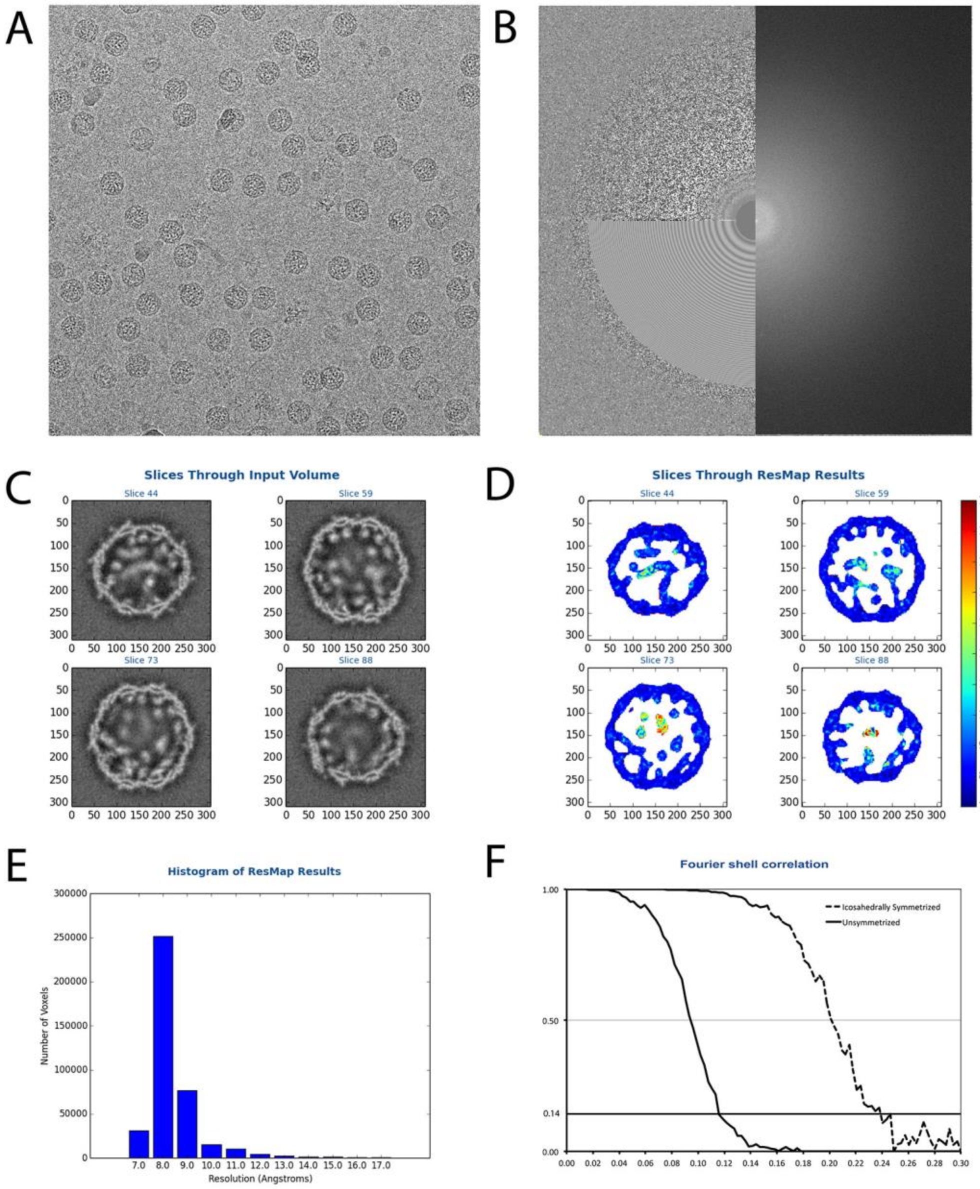

Supplementary Figure 2. Quality assessment of EM density maps.

Aligned and summed movie of vitrified MS2 particles (a) and FFT (left half background corrected) and CTFFIND fit for CTF correction (b). Slices through the asymmetric reconstruction (c), local resolution distribution in map (d), and resolution variability using ResMap (e) shows that resolution varies between 7 and 15 Å with high resolution parts (7 to 9 Å) localized in and near the capsid and with low resolution parts (10 to 15 Å) mainly localized in the central part of the reconstruction. Fourier Shell correlation of the unsymmetrized reconstruction and icosahedrally symmetrized reconstruction shows resolutions of resp. 8.7 Å and 4.1 Å using of the 0.14 cut-off criterion (f).

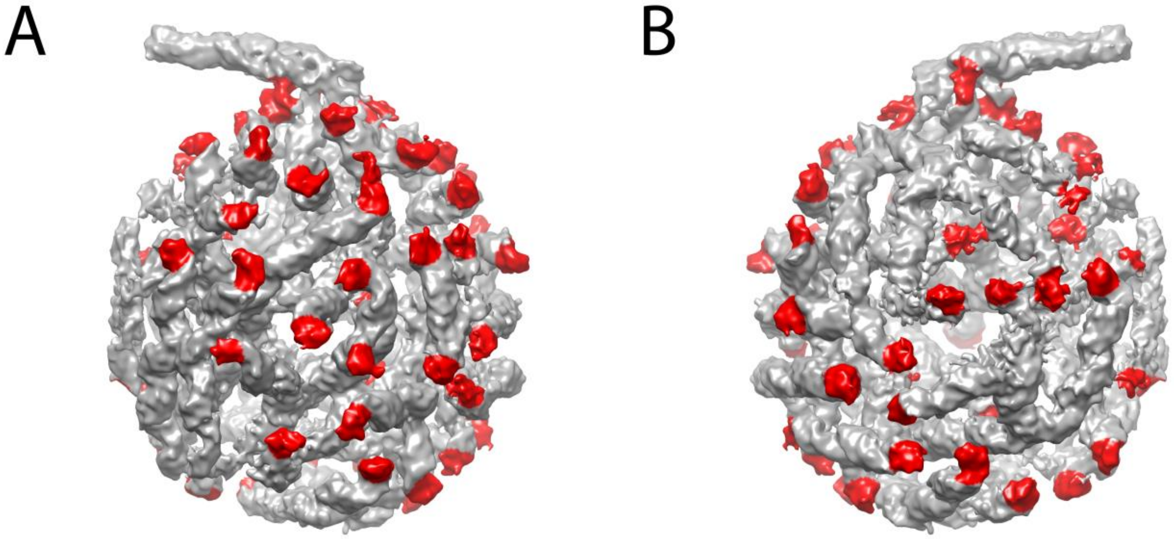

Supplementary Figure 3. Distribution of RNA stem loops.

Distribution of all SL ends in the left (a) and right (b) view of the MS2 RNA shows that the majority (53/59) is located near the capsid (red) and are unevenly distributed over the RNA-capsid interface.

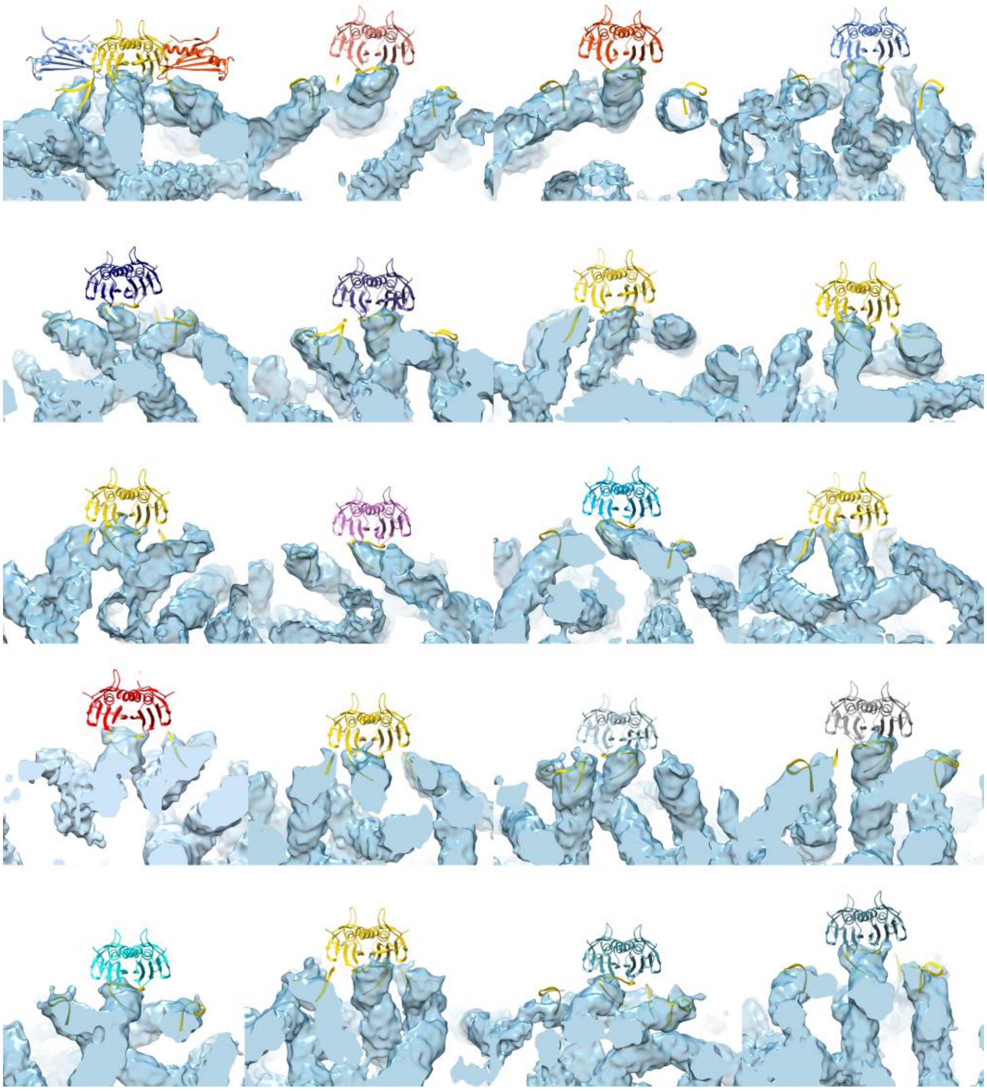

Supplementary Figure 4. Individual fits of RNA in EM density.

Twenty examples of the best fits of the TR (yellow) and accompanying CP_2 (several colours) in the EM density of the RNA (grey-blue).
